# Nano-based delivery of RNAi in cancer therapy

**DOI:** 10.1186/s12943-017-0683-y

**Published:** 2017-07-28

**Authors:** Yong Xin, Min Huang, Wen Wen Guo, Qian Huang, Long zhen Zhang, Guan Jiang

**Affiliations:** 1 Hospital of Xuzhou Medical University, Xuzhou, Jiangsu 221002 People’s Republic of China; 2 Department of Dermatology, Affiliated Hospital of Xuzhou Medical University, Xuzhou, 221002 China

**Keywords:** Nanocarriers, RNAi, siRNA, miRNA, Cancer therapy, Gene therapy

## Abstract

**Background:**

RNA interference (RNAi), a newly developed method in which RNA molecules inhibit gene expression, has recently received considerable research attention. In the development of RNAi-based therapies, nanoparticles, which have distinctive size effects along with facile modification strategies and are capable of mediating effective RNAi with targeting potential, are attracting extensive interest.

**Objective:**

This review presents an overview of the mechanisms of RNAi molecules in gene therapy and the different nanoparticles used to deliver RNAi molecules; briefly describes the current uses of RNAi in cancer therapy along with the nano-based delivery of RNA molecules in previous studies; and highlights some other carriers that have been applied in clinical settings. Finally, we discuss the nano-based delivery of RNAi therapeutics in preclinical development, including the current status and limitations of anti-cancer treatment.

**Conclusion:**

With the growing number of RNAi therapeutics entering the clinical phase, various nanocarriers are expected to play important roles in the delivery of RNAi molecules for cancer therapeutics.

## Background

Cancer is a major public health problem around the world [1], and the world-wide incidence of cancer continues to increase [2]. The world cancer rate is expected to double by 2020. The number of new cancer patients worldwide will reach 15 million a year. The primary treatments for cancer are surgery, chemotherapy and radiation therapy. Non-targeted treatments can result in negative side effects, which occur when the treatment affects healthy tissues or organs. These problematic side effects causes significant distress in patients and impede the course of cancer treatment. For this reason, a number of novel cancer treatments are currently under development, one of them being gene therapy.

During the last decade, remarkable advances have been made in genome research, revolutionizing the entire field. As a consequence, information may no longer be a bottleneck in understanding and tackling complex genetic diseases such as cancer [3], and gene therapy emerged as a method to treat cancer. RNA interference (RNAi) is one method of regulating target genes [4]. RNAi shows promise for the development of new classes of molecular therapeutic drugs that interfere with disease-causing or -promoting genes, particularly those that encode so-called “nondruggable” targets, which are not amenable to conventional therapeutics [5].

Although RNAi is thought to be more effective in treating disease compared to other methods [6], several challenges are associated with delivering small interfering RNAs (siRNAs) to diseased sites for gene therapy [7]. Two main approaches for the delivery of RNAi molecules have been developed: viral and non-viral vectors. However, nanoparticles have recently received attention for use in RNAi. The paradigm shift to the use of nanoparticles for RNAi molecules delivery is attributed to unique benefits provided by nanoparticles in comparison to other carriers.

In this review, we primarily discuss the delivery of RNAi molecules by nanoparticles in cancer therapy. We hope that this review provides useful information to help translate this novel therapy to clinical application.

## Main text

### Types of RNAi molecules

**Fig. 1:**
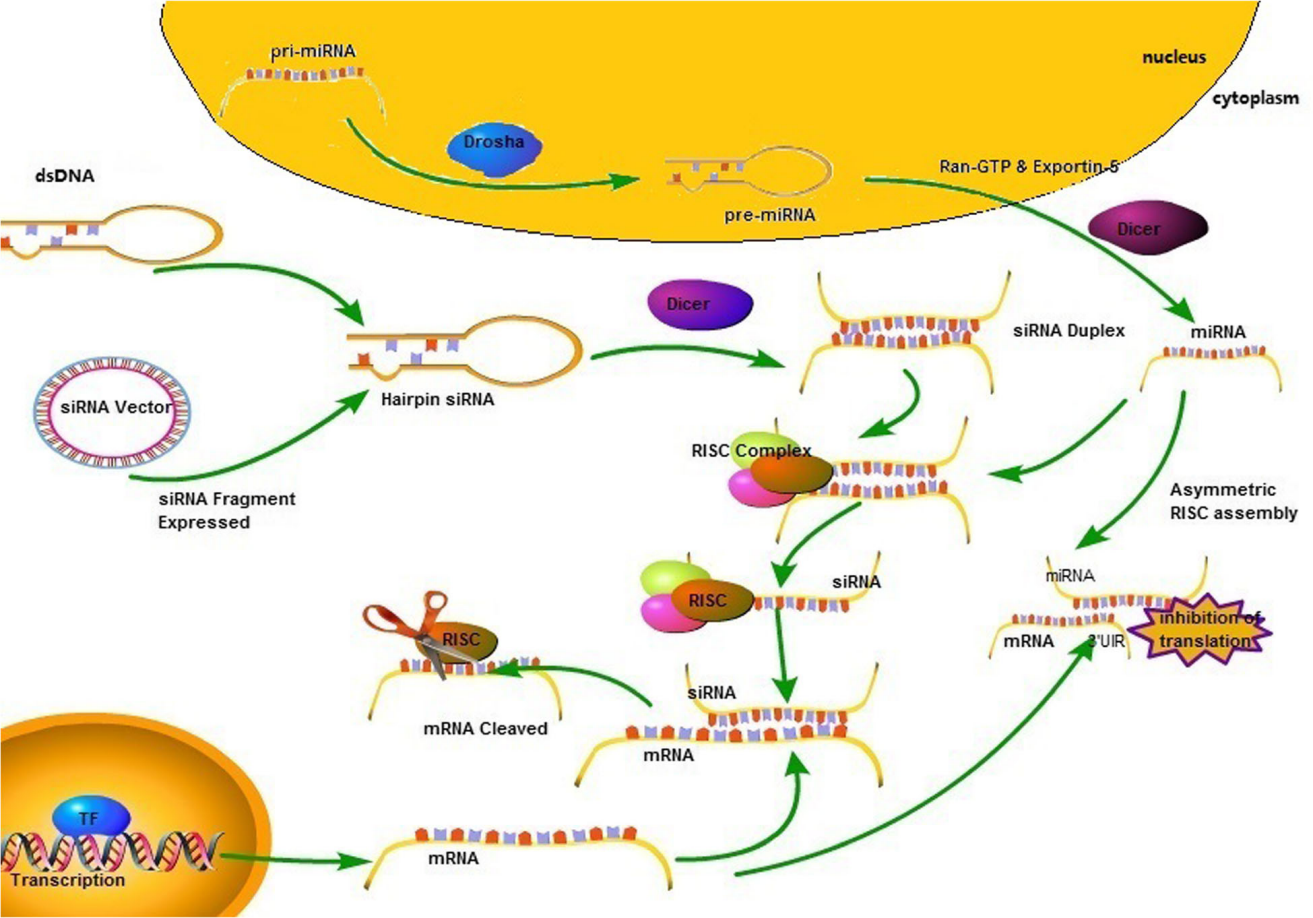
Mechanism of RNA interference (RNAi). A pri-miRNA molecule is processed by an RNAse III enzyme called Drosha and becomes pre-miRNA. Exportin 5 exports the pre-miRNA into the cytoplasm where it is processed by the RNAse III enzyme Dicer and becomes the mature miRNA. After this point, the endogenous pathway is similar to the exogenous pathway, in which synthetic RNAi molecules are inserted into the cell cytoplasm. The double-stranded RNA molecule with 19–23 base pairs is then incorporated into RISC (or miRISC), were the passenger strand is released, and the guide strand mediates the degradation or translation inhibition of its target mRNA

The different types of RNAi molecules are microRNA (miRNA), siRNA and short hairpin RNA (shRNA). In RNAi, RNAi molecules delivered into cells initiate the degradation of complementary messenger RNA (mRNA) molecules via the cells’ internal machinery. This halts the production of the proteins encoded by the mRNAs, resulting in reduced gene expression (Fig. 1) [8].

siRNAs show great potential for use in nucleic acid therapeutics because of their potent and specific RNAi-triggering activity [9]. siRNA is a chemically synthesized, double-stranded RNA (dsRNA) containing 19–23 base pairs with 2-nucleotides unpaired in the 5′-phosphorylated ends and unphosphorylated 3′-ends [10]. SiRNAs are incorporated into an RNA-induced silencing complex (RISC), a protein-RNA complex that separates the strands of the RNA duplex and discards the passenger (sense) strand. The guide (anti-sense) strand then guides the RISC to anneal and cleave the target mRNA or block its translation [11].

miRNAs are endogenous non-coding RNAs involved in the post-transcriptional regulation of gene expression [12]. They are produced by a similar mechanism as siRNAs from longer precursor molecules (pre-miRNA), which are transported to the cytoplasm, and are also substrates for Dicer [13]. There, RNAse III Dicer generates dsRNA containing approximately 22 nucleotides (i.e., the mature miRNA). The miRISC usually hybridizes to partially complementary binding sites on the 3-untranslated regions of the target mRNAs [14]or combines with targeted mRNA and promotes its cleavage [15].

In addition to siRNA and miRNA, shRNA can also be cloned into an expression vector and express siRNA. This makes it possible to structure an RNAi carrier to silence intracellular gene expression in mammals. shRNAi can use RNA polymerase III-dependent promoters (small nuclear RNA U6 or RNaseP) to realize RNAi. The transformation of shRNA can cause long-time gene silence in mammals. Ohno et al. developed a 30-nucleotide, single-strand RNA termed guide hairpin RNA (ghRNA) that has a similar physiological function similar to miRNA and siRNA and causes no innate cytokine response in vitro or in vivo [16].

siRNA, miRNA and shRNA are all RNA molecules that rely on the Dicer enzyme; they can mediate RNAi and lead to the cleavage of mRNA. miRNA is single-stranded RNA, whereas siRNA and shRNA are double-stranded RNAs. The fundamental difference between siRNA and miRNA is that miRNA is endogenous (i.e., miRNA is an inherent component of the organism), whereas siRNA is artificially synthesized and transfected into the human body, where it operates within the RNAi pathway. miRNA primarily acts on the 3′-untranslated region of the target gene, while siRNA can act on any part of mRNA [17]. miRNA is produced asymmetrically, whereas siRNA is symmetrically derived from the two side arms of the front body of double-chain RNA. shRNA can be cloned into carriers that then express siRNA.

## RNAi and cancer

### Therapeutic implications

The activation of proto-oncogenes such as chromosomal rearrangements, insertion mutations, point mutations, and gene amplification [18] can tip the balance between proto-oncogenes and tumor-suppressing genes, which can result in cancer [19]. The different types of activated oncogene mRNA can be effectively inhibited by RNAi technology to inhibit tumor growth. Wilda et al. reported that transfecting m-bcr/abl siRNA to the chronic myelogenous leukemia K562 cell line inhibited the expressions of m-bcr/abl mRNA and protein, decreased the degree of cell malignancy, and induced cell apoptosis [20].

There is a certain relationship between the role of the gene expression and apoptosis genes. RNAi knockout technology plays a role in the inhibition of the occurrence and development of a variety of tumors. Cioca et al. reported that a specific combination of Bcl-2 and c-raf of the siRNA gene can induce apoptosis in HL-60, U937 and THP-1 leukemia cell lines and enhance their sensitivity to etoposide and daunorubicin [21]. This indicates that RNAi molecules can improve the efficiency of chemotherapy. MDR1 is a multidrug-resistance gene that plays an important role in tumor chemotherapy resistance. Nieth et al. found that transfecting MDR1 siRNA into pancreatic cancer and gastric cancer cell lines significantly inhibited MDR1 mRNA and protein and reduced the resistance to daunorubicin [22]. This indicates that RNAi can be used to reverse multidrug resistance and restore sensitivity to chemotherapeutic drugs.

**Fig. 2:**
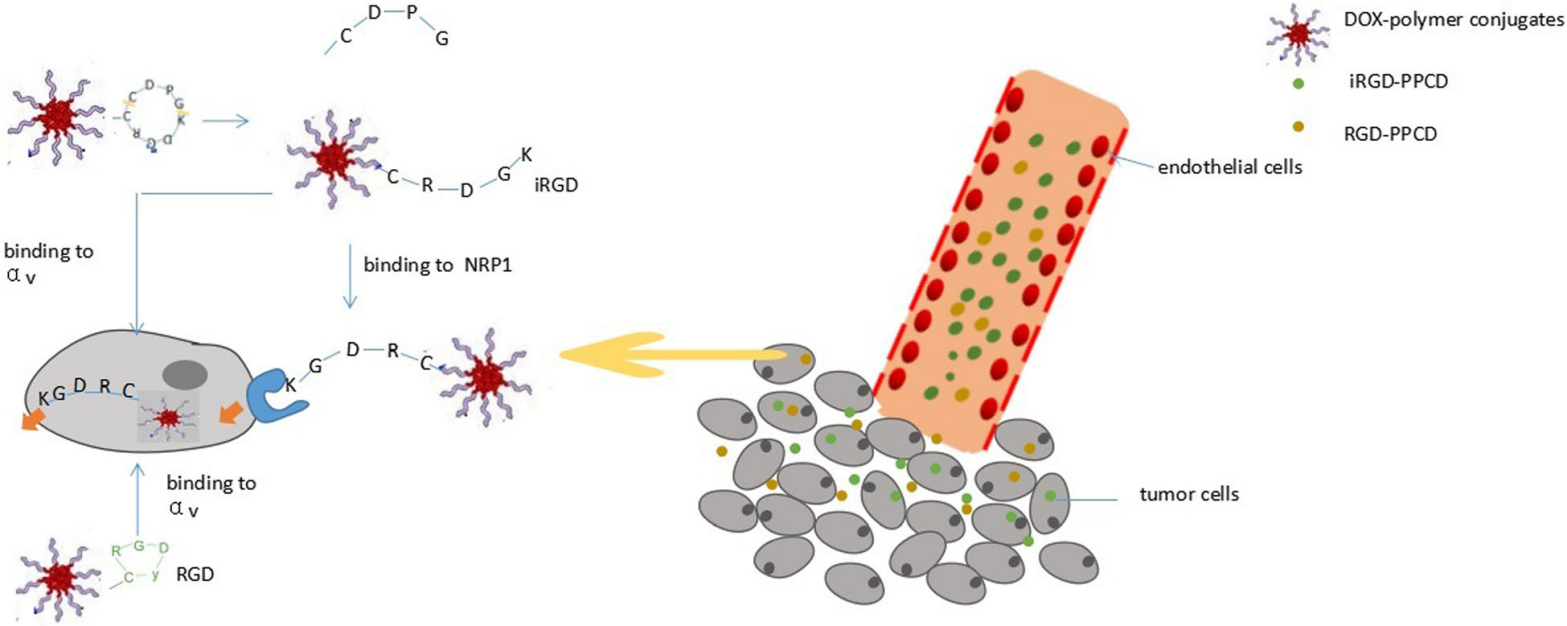
Illustration of in vivo delivery and tumor penetration of iRGD-PPCD and RCD-PPCD. iRGD cyclopeptide (iRGDyC) was bound to PAMAM dendrimer with DOX by an acid-sensitive cis-aconityl linkage (PEG-PAMAM-cisaconityl DOX, PPCD), producing an iRGD–PPCD conjugate. The iRGD–PPCD conjugate is proteolytically cleaved by the proteinase on the cell surface to generate a C-terminal PPCDCRGDK sequence capable of binding to neuropilin-1 and providing a transport pathway out of the blood vessels and through the extracellular tumor tissue. In contrast, RGD–PPCD only accumulates around the tumor blood vessels by binding to alpha v integrins and shows poor penetration ability in the tumor parenchyma

In addition, nanocarriers can be further functionalized with targeting moieties based on the target organ using polyethylene glycol (PEG) and/or other ligands [23]. Wang et al. [24] bound internalizing RGD peptide (a tumor-targeting and tumor-penetrating cyclic peptide; amino acid sequence: CRGDKGPDC) to PEGylated polyamidoamine (PAMAM) dendrimer with doxorubicin (DOX) by acid-sensitive cis-aconityl linkage (PEG-PAMAM-cisaconityl-DOX, PPCD) and confirmed that iRGD-mediated PPCD had obvious advantages. These advantages were mainly reflected in the increased tumor vascular permeability, inhibition of tumor vascular growth, enhancement of intratumoral drug accumulation, and the resulting longer survival time (Fig. 2). Because of these advantages, different nanoscale delivery systems have been used to apply RNAi in cancer therapy [25].

Gene-knockout technology plays a role in cancer cell growth, proliferation, metastasis, and multi-drug resistance. Recent studies have identified several cancer-related genes as potential targets for RNAi-based therapy.

### Difficulties in application

Since the expression and functionality of siRNAs and miRNAs are significantly reduced in cancer cells, RNAi is an attractive target for the development of innovative therapeutics. However, the main obstacle to achieving gene silencing in vivo using RNAi techniques is the delivery of therapeutic RNAi molecules [26]. Thus, the clinical application of RNAi therapy faces many challenges, including supplying specific gene targets to the appropriate tissue and cell types at safe and effective dosages and maintaining oligonucleotide stability in circulation. Strategies for enhancing cellular uptake and methods for monitoring the distribution and therapeutic efficacy are also needed [27–29].

### Nanoparticles applied to deliver RNAi molecules

Recently, nanoparticles have received considerable attention as vectors for gene delivery [30]. Nanoparticles are particulate dispersions or solid particles with particle sizes in the range of 10–1000 nm [31]. One advantage of using nanoparticles for delivery is the enhanced permeability and retention (EPR) effect, which enables nanocarriers to accumulate in tumors at much higher concentrations than in normal tissues [32]. Nanocarriers can protect RNAi molecules from enzymatic degradation and immune recognition, have much higher transportation efficiency across the cell membrane compared to other carriers [33], and can prevent excretion if the carrier size and surface coating are appropriate [34]. The constituents of nanocarriers applied to RNAi can be classified as organic nanoparticles and inorganic substances (Table 1).

**Table 1:** Characteristics of different nanoparticles

Nanocarriers	Classification	Different features	Common features
Polymer-based nanoparticles	Synthetic polymers: PEI, PLL, PAMAM; natural polymers: chitosan, atelocollagen	Higher thermodynamic stability and dynamic stability	1. Low toxicity, good biocompatibility, high security 2. Biodegradable, low immunogenicity 3. Nanocarrier surfaces should be positively charged 4. High gene transfection efficiency 5. Mass production, low cost
Lipid-based nanoparticles	Liposomes, micelles, emulsions, solid lipid nanoparticles	Superior stability; can be sterilized and freeze-dried	
Inorganic nanoparticles	MSNs, carbon nanotubes, QDS, gold nanoparticles	High surface area and large pore volume, strong surface plasmon resonance absorption	
Exosome-mimetic nanovesicles		High production yield, efficient loading and reduction of target cells	

Organic nanoparticles include cationic polymer nanoparticles, lipid-based systems and so on. Drugs can be incorporated into organic nanoparticles via chemical bonding or physical embedding [35]. Among the various cationic polymers, synthetic polymers [e.g., polyethylenimine (PEI), poly-L-lysine (PLL), and cyclodextrin-based polycations] and natural polymers (e.g., chitosan and atelocollagen) have been used as carriers. PEI is a widely studied cationic polymer for the delivery of RNAi. However, PEI has failed to progress clinically, primarily owing to its poor toxicity profile and in vivo instability, particularly during systemic administration [36]. PEG has been used to reduce the cytotoxicity of PEI. For example, Kim et al. [37] developed PEI/PEG-conjugated siRNA polyelectrolyte complexes; these PEGylated polyplexes containing vascular endothelial growth factor (VEGF) siRNA were accumulated in tumor regions, and the knockdown of VEGF suppressed microvessel formation, thereby inhibiting tumor growth.

Various lipid-based systems have been reported, including liposomes, micelles, emulsions, and solid lipid nanoparticles (SLNs). Generally, cationic lipids have been used as siRNA delivery carriers because they associate easily with negatively charged nucleic acids [38]. The physicochemical properties of lipid-based nanoparticles (e.g., the structure, size, and surface charge) may be optimized by modifying the lipid composition, drug-to-lipid ratio, and manufacturing process. Recently, SLNs with sizes in the range of 10 to 1000 nm have been utilized for the delivery of RNAi because they can be sterilized and freeze-dried because of their superior stability in the human body [39].

More and more inorganic substances, such as mesoporous silica nanoparticles (MSNs), carbon nanotubes, quantum dots and metal nanoparticles, are being reported as carriers for RNAi deliver. Inorganic nanomaterials are synthesized by inorganic particles and biodegradable polycation. They always play a role in gene therapy by a novel nanosize drug carrier [40]. Among inorganic nanocarriers, MSNs are the most commonly used because of their unique properties such as uniform mesopores, easy functionalization, biocompatibility, high surface area, large pore volume, and biodegradability [41, 42]. To enhance the siRNA loading efficiency and the cellular uptake efficacy of MSNs, the surfaces or inner pores of MSNs have been coated with cationic polymers. For example, Li et al. loaded siRNA in the inner pores of MSNs and then coated the nanoparticle surfaces with the cationic polymer PEI and fusogenic peptide KALA (a cationic peptide consisting of 30 amino acids). The delivery of siRNA inhibited the expression of VEGF protein and tumor angiogenesis, thereby leading to the suppression of tumor growth [43].

Gold nanoparticles (AuNPs) have received great interest in biomedical applications. For siRNA loading, AuNP can be directly conjugated to siRNA via gold-thiol chemistry. Conde et al. [44] functionalized AuNPs with siRNA as a therapeutic agent and arginylglycylaspartic acid (RGD) peptide to target lung cancer. Furthermore, a single-chain variable fragment targeting epidermal growth factor receptor (EGFR), transferrin, and an aptamer targeting a prostate-specific membrane have been used to impart AuNPs with the ability to target cancer cells [45–47].

In a recent study, exosome-mimetic nanovesicles showed high production yield and the ability to deliver anti-cancer chemotherapeutics into cells [48]. Lunavat et al. identified several advantages of exosome-mimetic nanovesicles, including high yield during production compared to exosomes [49] and the possibility of producing them from engineered cells expressing specific surface molecules [50, 51]. Thus, exosome-mimetic nanovesicles represent valuable vehicles for the delivery of RNAi, possibly for the treatment of different types of diseases [49]. In their study, Lunavat et al. [52] showed that exosome-mimetic nanovesicles are efficiently loaded with siRNA, both when introduced to the nanovesicles exogenously and when produced endogenously. In addition, the reduction of target genes was efficient for both loading methods.

### Studies on the nano-based delivery of RNAi molecules

The nanoparticle-based delivery of RNAi molecules can produce multiple advantages. Many studies (Table 2) have identified the efficient applications of RNAi delivered by diverse nanocarriers.

**Table 2:** Summary of studies on the nano-based delivery of RNAi molecules

RNAi	Nanocarriers	Targeted cell lines	Tissue	Reference
siRNA	PEI-PEG	SGC7901	gastric cancer cells	[53]
	Poly-L-lysine nanoshells	H1299	lung cancer cells	[54]
	Single-walled carbon nanotubes	MCF-7	breast cancer	[55]
miRNA	DSS-BEN	HCT-116	colorectal cell	[58]
	AuNPs	MM.1S	multiple myeloma cells	[59]
shRNA	Alkylmodified polyethylenimine	MCF-7	breast cancer	[60]

Wu et al. concluded that PEI-PEG may be a promising non-viral carrier for altering gene expression in the treatment of gastric cancer due to several advantages including relatively high gene transfection efficiency and low cytotoxicity; their previous research demonstrated that PEI-PEG can efficiently deliver RNAi to SGC7901 gastric carcinoma cells and suppress CD44v6 expression [53].

Huschka et al. quantitatively demonstrated the remotely controlled, light-triggered release of antisense ssDNA from poly-L-lysine nanoshell vectors [54]. This poly-lysine nanoshell complex was successfully used as a nonviral delivery vector that could controllably release antisense oligonucleotide ssDNA and siRNA in vitro. Their findings indicate that this nanoparticle complex can further be used to quantify the number of molecules delivered intracellularly, which could have extensive applications in studying the rates of specific processes within living cells.

Mohammadi et al. developed a novel nanoparticle based on single-walled carbon nanotubes conjugated to piperazine–PEI derivatives for breast cancer gene therapy. This nanoparticle targets epithelial cell adhesion molecules, which are frequently overexpressed in solid tumors and were recently identified as a cancer stem cell marker [55]. The vector–aptamer conjugate could efficiently increase DNA transfection. The structural modification(amide bond formation between single-walled carbon nanotubes and PEI) employed in their study also reduced cytotoxicity compared with PEI 25 kDa.

In addition to siRNA, many studies have been carried out on the roles of miRNA and shRNA in cancer therapy. Human cancer was recently associated with changes in miRNA profile [56], drawing even more attention to the nanocarrier-based delivery of miRNA.

A biodegradable polycationic prodrug named DSS-BEN was synthesized from the polyamine analog N^1^,N^11^-bisethylnorspermine and selectively disassembled in the cytoplasm, where it released miRNA [57]. Xie et al. developed an innovative combinatorial strategy for treatment based on the simultaneous regulation of polymer prodrug nanoparticle. They hypothesized that simultaneous deliveryof miR-34a and BENSpm by DSS-BEN/miR-34a nanoparticles will lead to improved combination antitumor activity. The results showed enhanced cell killing in vitro along with improved tumor growth inhibition in HCT116 xenografts in vivo [58].

An experiment was carried out to observe the inhibition of human multiple myeloma cells based on conjugation of gold nanoparticles with miRNAs [59]. The resulting miRNA − AuNPs were shown to exhibit effective transfection in multiple myeloma cells. These findings have implications for the design of miRNA-conjugated gold nanoparticles as effective biosensing and targeting probes.

Ebrahimian et al. fabricated a novel nanoparticle formulation based on poly lactic-coglycolic acid (PLGA) for the simultaneous delivery of small amounts of DOX and bcl-xl shRNA [60]. In vitro assessment revealed that PLGA-DOX-alkyl-PEI/pBcl-xL shRNA with sustained release kinetics could induce mortality to tumor cells and inhibit breast cancer proliferation better than free DOX and PLGA-DOX-PEI/pBcl-xL shRNA, indicating that the combination of PLGA-DOX NPs and alkyl-PEI/shRNA complexes may have promising applications in breast cancer therapy.

### **Other carriers applied to transfer RNAi molecules** (Table 3)

RNAi has great potential for therapeutic treatments. Although siRNA has been researched for a long time, miRNA has only recently generated significant attention, and many gaps in knowledge remain. In clinical settings, there are many obstacles to the targeted delivery of siRNA in vivo, including the specificity and stability of the RNAi reagents [61]. In recent years, progress has been made to enhance the efficacy and specificity of lipids, nanoparticles, polymers, bacteria, and viral vectors used as delivery agents while simultaneously reducing their toxicity [62].

**Table 3:** Comparison of different types of carriers for RNAi molecules

Carrier	Manner	Key characteristics	Common advantages
Viral vectors	Integrated into the host chromosome	Suitable for splinter cells and the cells that are difficult to transfect; can transfect targeting cells naturally and efficiently	1. Improve the effectiveness 2. Improve the specificity 3. Lower toxicity
Lipids	Conjugate or complexing	High drug entrapment efficiency; adjustable continuous release behavior and good serum stability; ability to protect the medicine from degradation; no immunogenicity	
Polymers	Combined with the skeleton phosphate or connected to the adapter body	Low transfection efficiency	
Bacteria	Specific antibodies	Non-toxicity, lack of side effects, can be manipulated	

Viral vectors are optimal vehicles for gene transfer because of their ability to efficiently infect host cells [63]. At present, the most commonly applied virus vectors are retroviral, adenovirus, adenovirus-associated, slow virus, and herpes simplex virus vectors. Each virus has its own advantages [64]. For example, retroviral and lentiviral vectors can integrate into the host genome, leading to long-term gene expression even after a single administration. Adenoviral vectors can efficiently transduce both dividing and non-dividing cell types, although they may cause immunostimulation, which often limits their in vivo application. Finally, adeno-associated viruses can also infect many non-dividing and dividing cell types, but have limited capacity for DNA insertion [65–67].

Lipids are promising and versatile carriers because they can be custom designed to have functional properties that allow for the protection of siRNA, steric stabilization, targeting, membrane destabilization and triggered drug release [68]. However, lipids still require more development as carriers for RNAi [69]. The encapsulation efficiencies of lipid-based liposomes used to deliver siRNA are not optimal; that is, many siRNA molecules remain free in solution. To decrease the amount of free siRNA, the number of liposomes must be increased; however, this causes additional toxicity both in vitro and in vivo. Another remaining challenge is the control of the morphologies and sizes of lipoplexes [70].

Peptides can replace liposomes for the delivery of siRNA to some cells; histidine-lysine (HK) polymers have been reported to be more effective than liposomes in the delivery of siRNA to targeted cells. Lysine in HK polymers can combine with the phosphate backbone of siRNA, while histidine increases the release of siRNA in vivo by acting as a proton pump; these two peptides cooperate with each other to accomplish the delivery of RNAi. Additionally, polymers can connect to the adapter body and deliver siRNA to targeted cells. With their biodegradability and diversity of permutations, peptides have significant potential as carriers of nucleic acids. However, their low transfection efficiencies mean that peptides must be modified by PEI [71], which can lead to toxicity. PEG modification can decrease the toxicity.

Bacterial colonization in tumors has been recognized to have a beneficial effect in cancer cells [72]; these microorganisms naturally accumulate and replicate in a variety of solid tumors, leading to reduced tumor size. Bacteria, bacteriophages, and bacteria-like particles such as minicells have been investigated as agents for gene delivery. Using bacteria for siRNA therapy provides yet another approach for the safe delivery of siRNA and has been shown to effectively silence genes without toxic side effects. To customize the use of bacteria for the treatment of various diseases in a variety of tissues, the bacteria can be manipulated, or different strains can be used. Based on these characteristics, the bacterial delivery of siRNA is an up-and-coming approach for RNAi therapy [73].

Many studies have been carried out in recent years to discuss the specific roles of miRNA in health and disease [74, 75]. Nevertheless, these data still have not been incorporated into therapeutic tools. In the near future, it is likely that existing strategies for siRNA delivery will also be exploited for the therapeutic delivery of miRNA mimetics and antagomirs [76].

## Conclusions and perspectives

RNAi is an economic, fast, and efficient method of cancer therapy. Many studies have shown that siRNA has a significant therapeutic effect on cultured tumor cells; however, few in vivo studies have been reported because siRNA is easily degraded in vivo, the transfection efficiency and targeting are low. Nanoparticles have been successfully demonstrated as efficient carriers for the delivery of RNAi molecules due to their unique characteristics. The addition of active targeting molecules to nanoparticles can enhance the tumor-targeting efficacy or the transfection efficiency to tumor cells. However, nanoparticle carriers still possess disadvantages that limit their application, including immunogenicity and toxicity. Furthermore, the detailed mechanism of RNAi is not completely clear, and the identification of RNAi molecules’ targets and the signaling pathways involved in the regulation of RNAi molecule expression in tumor formation remains a challenge. Although RNAi molecules are effective in silencing oncogenes, it is critical to identify the RNAi regulatory network rather than just a few isolated targets. Thus, more studies on the delivery of RNAi molecules by nanoparticles in cancer therapy are needed to establish a new method of treating cancer.

## Abbreviations

AuNPs: Gold nanoparticles
DOX: Doxorubicin
dsRNA: Double-strainded RNA
EGFR: Epidermal growth factor receptor
EPR: Enhanced permeability and retention
ghRNA: Guide hairpin RNA
HK: Histidine-lysine
miRNA: microRNA
mRNA: Messenger RNA
MSNs: Mesoporous silica nanoparticles
PAMAM: PEGylated polyamidoamine
PEG: Polyethylene glycol
PEI: Polyethylenimine
PLGA: Poly lactic-co-glycolic acid
PLL: Poly-L-lysine
pre-miRNA: microRNA precursors
RGD: Arginylglycylaspartic acid
RISC: RNA-induced silencing complex
RNAi: RNA interference
shRNA: Short hairpin RNA
siRNAs: Small interfering RNAs
SLNs: Solid lipid nanoparticles
VEGF: Endothelial growth factor
